# Tissue-engineered anterior segment eye cultures demonstrate hallmarks of conventional organ culture

**DOI:** 10.1007/s00417-022-05915-z

**Published:** 2022-12-24

**Authors:** Susannah Waxman, Alicja Strzalkowska, Chao Wang, Ralitsa Loewen, Yalong Dang, Nils A. Loewen

**Affiliations:** 1 Department of Ophthalmology, University of Pittsburgh School of Medicine, Pittsburgh, PA USA; 2 Department of Ophthalmology, University of Würzburg, Würzburg, Germany; 3 Eye Center of Xiangya Hospital, Central South University, Changsha, Hunan China; 4 Sanmenxia Central Hospital, Sanmenxia, Henan China; 5 Artemis Eye Centers of Frankfurt, Hanauer Landstr. 147-149, 60314 Frankfurt, Germany

**Keywords:** Ocular anterior segment perfusion culture, Tissue engineering, Aqueous humor outflow, Trabecular meshwork

## Abstract

**Background:**

Glaucoma is a blinding disease largely caused by dysregulation of outflow through the trabecular meshwork (TM), resulting in elevated intraocular pressure (IOP). We hypothesized that transplanting TM cells into a decellularized, tissue-engineered anterior segment eye culture could restore the outflow structure and function.

**Methods:**

Porcine eyes were decellularized with freeze–thaw cycles and perfusion of surfactant. We seeded control scaffolds with CrFK cells transduced with lentiviral vectors to stably express eGFP and compared them to scaffolds seeded with primary TM cells as well as to normal, unaltered eyes. We tracked the repopulation behavior, performed IOP maintenance challenges, and analyzed the histology.

**Results:**

Transplanted cells localized to the TM and progressively infiltrated the extracellular matrix, reaching a distribution comparable to normal, unaltered eyes. After a perfusion rate challenge to mimic a glaucomatous pressure elevation, transplanted and normal eyes reestablished a normal intraocular pressure (transplanted = 16.5 ± 0.9 mmHg, normal = 16.9 ± 0.9). However, eyes reseeded with eGFP-expressing CrFK cells could not regulate IOP, remaining high and unstable (27.0 ± 6.2 mmHg) instead.

**Conclusion:**

Tissue-engineered anterior segment scaffolds can serve as readily available, scalable ocular perfusion cultures. This could reduce dependency on scarce donor globes in outflow research and may allow engineering perfusion cultures with specific geno- and phenotypes.

**Supplementary Information:**

The online version contains supplementary material available at 10.1007/s00417-022-05915-z.


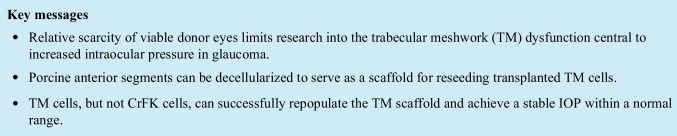


## Introduction

Glaucoma is a progressive optic neuropathy that affects over 70 million people worldwide and can cause irreversible blindness [1]. Clear fluid in the eye’s anterior chamber, called aqueous humor, is produced within the confines of the globe at 2 to 3 µL/min [2, 3]. In healthy eyes, aqueous humor outflow is in equilibrium with production, but in primary open angle glaucoma, an increased outflow resistance elevates the intraocular pressure (IOP) [4, 5]. Intentionally elevating IOP in primate studies leads to glaucoma, while lowering it prevents it [6]. IOP remains the only clinically relevant factor that can be altered to reduce glaucomatous retinal ganglion cell death and vision loss [7, 8]. As a primary site of outflow pathogenesis, the trabecular meshwork (TM) is a target of great therapeutic interest. The TM is a dynamic, multilayer, filter-like structure that responds to environmental signals like mechanical strain and shear forces with cytoskeletal and extracellular matrix (ECM) changes to maintain a normal IOP [9–13]. In glaucoma, the TM is characterized by senescence [14, 15], increased TM stiffness [16], and reduced cellularity [17].

The replacement of TM cells has therapeutic potential but could also serve as a new ex vivo model to study diseased or intentionally altered cells. Simple in vitro TM cultures lack a pressure gradient and flow, defining features of both healthy and glaucomatous physiology. In vitro, TM perfusion models that use thin, layered scaffolds have been constructed to address these shortcomings [18, 19]. However, these models can still not replicate the complex 3D structure of ECM beams and fibers of native TM. Their translational impact is often limited by cell physiology that is different compared to a complex organotypic substrate, which strongly resembles normal in vivo physiology [20–23]. Ex vivo perfused anterior segment models from a variety of species have been developed to examine and manipulate TM function [24–30] because there is a scarcity of whole human donor eyes for research. In contrast to this shortage, TM cells from corneal rims used for corneal transplantation, TM-removal surgery, and TM cell lines are more readily available [31, 32] and could lend themselves to scalable studies when expanded and seeded into decellularized anterior segment scaffolds from pig eyes. As done in other tissues in research [33–38] and medical applications [39–42], the removal of cells from ECM can provide a bioartificial scaffold for recellularization with cells of choice. We had previously described a freeze–thaw protocol for this purpose [43], but in pilot experiments detected cell debris when we developed a protocol for recellularization. The surfactant sodium dodecyl sulfate (SDS) has been used to remove cellular material in various ECM-rich tissues, while hallmark TM ECM components, like collagen, elastin, and laminin, were mostly well conserved [33, 44–46].

We hypothesized that decellularized porcine anterior segments [43] could be repopulated with non-native TM cells to maintain IOP within a physiologic range. We seeded the eyes with porcine TM cells in this feasibility study to use fresh cells with high viability. The porcine anterior segment scaffolds described here are accessible, storable, biocompatible, free of porcine cells, and can be seeded with transplanted cells.

## Methods

A total of 57 anterior segments were prepared as previously described [24, 47, 48] (Supplemental material 1). Briefly, porcine eyes were acquired within 4 h of sacrifice, decontaminated with povidone-iodine, and hemisected along the equator in an aseptic biosafety cabinet. The posterior segment, lens, and iris were carefully removed.

### Scaffold production

Freeze–thaw-treated [43, 49] anterior segments (FT) were sealed in an airtight container and cycled between − 80 °C and room temperature two times to lyse all native cells [43, 49]. Freeze-thawed scaffolds were perfused with culture media (DMEM supplemented with 1% FBS and 1 × antibiotic/antimycotic) for 5 days (*n* = 4). A time-course assay was conducted to determine the minimum time to produce decellularized scaffolds via immersion and agitation-mediated decellularization (IA). Freeze-thawed anterior segments were placed in SDS solution (0.01% wt/vol in PBS + anti-anti) for 1 day (*n* = 3), 2 days (*n* = 3), and 5 days (*n* = 3). Following SDS incubation, solutions were changed to 0.1% TritonX-100 for 24 h and a perfusion culture media wash for 48 h. Samples were affixed to a vertical stage rotating at 20 RPM during each incubation. Fluid exchanges were performed every 24 h.

For perfusion-mediated decellularization (P), we modified an existing matrix production protocol used in bioartificial heart construction [33]. Freeze-thawed segments were maintained via constant-rate perfusion [25] at 6 µL/min with SDS solution for 24 h, TritonX-100 solution for 24 h, and perfusion culture media for 48 h (*n* = 24). Untreated control samples were maintained for 5 days with perfusion culture media (*n* = 4). All scaffolds were stored at − 80 °C and up to 2 months before reseeding. To avoid any confounding effects from prior experiments, none was reused.

### Cell culture

Porcine TM culture was performed as done previously [24, 50]. The TM was dissected away from anterior segments under an ophthalmic operating microscope (Stativ S4, Carl Zeiss, Oberkochen, Germany) and cut into 0.5-mm^3^ segments. Tissue pieces were cultured in T25 flasks containing OptiMEM (31,985–070, Gibco, Life Technologies, Grand Island, NY, USA), supplemented with 5% FBS and antibiotic/antimycotic (15,240,062, Thermo Fisher Scientific, Waltham, MA, USA). The method used here to obtain cells from the porcine angular plexus naturally provides a non-homogeneous cell population. Cells were passaged at 80% confluence and used for experiments at passages 2–4.

For initial scaffold biocompatibility testing and real-time tracking of seeded cells, we used a versatile CrFK cell line (CRFK ATCC CCL-94) transduced with the eGFP-expressing feline immunodeficiency viral (FIV) vector GINSIN [25, 51]. Cells were transduced at a multiplicity of infection (MOI) of 5 transducing units (TU) per cell and enriched by fluorescence-activated cell sorting (FACS) after 7 days with a high fluorescence gate cut-off that eliminated all non-fluorescent cells. GINSIN-transduced CrFK cells were maintained in 2.5% FBS DMEM with antibiotic and antimycotic and passaged at 80% confluence.

### Live-cell tracking

Three million GINSIN-transduced CrFK cells were seeded into scaffolds to determine biocompatibility and cell localization in real-time. A 20G cannula (BD PrecisionGlide 305,176) attached to a 1-mL syringe (Norm-Ject tuberculin Luer 4010-200V0) was connected to a 3 cm length of perfusion tubing. The inlet and outlet of each perfusion dish were disconnected from the perfusion system and reconnected to the 1-mL syringes. A 200 µL bolus of culture media was removed from the anterior chambers through the outlet. A 200 µL bolus of 3 million GINSIN-CrFK cells was slowly introduced into the anterior chamber. Cultures were gravity perfused at 15 mmHg for 20 m. Dishes were positioned with corneas facing downward, and perfusion culture was restarted.

TM was visualized from the underside of transparent perfusion dishes with an epifluorescence-equipped dissecting microscope (Olympus SZX16 with GFP filter cube and DP80 monochrome/color camera; Olympus Corp., Center Valley, PA, USA) and imaged at 24, 48, and 144 h post-seeding. Mean fluorescent intensity of images was measured in four concentric regions (central cornea (1), peripheral cornea (2), TM (3), and sclera/ciliary body remnant (4), Fig. 2, bottom right) in Fiji [52] and compared. A uniform threshold was applied to fluorescence images to aid visualization in Fig. 2, right panel.

### IOP maintenance challenge

Decellularized scaffolds were perfused for 24 h with TM culture media before seeding to achieve stable baseline IOPs. Three million porcine TM cells were seeded as described above in RS (*n* = 8) while D (*n* = 8) received a sham bolus of cell-free TM culture media. Normal anterior segment cultures were used as controls (*n* = 8). After 48 h, the infusion rate was doubled from 3 to 6 µL/min to challenge the TM’s IOP maintenance response.

### Histology

Samples were fixed in 4% PFA for 48 h, embedded in paraffin, and sectioned at 6 µm thickness. Sections were stained with hematoxylin and eosin (H&E) for morphological analysis and DAPI for DNA content. Specimens were analyzed from the superior and inferior portions of each anterior segment.

### Statistical analysis

Fluorescence intensities of DAPI-labeled scaffold samples were compared to control. Live-cell eGFP fluorescence of the TM region was compared to all other locations. Cell depth at each time-point was compared to control. As in our prior anterior segment perfusion organ culture studies [24, 48, 53–56], perfusion system operators were trained to use the perfusion system under the same instructor (N.A.L.) and with the same detailed documentation. Work was checked by multiple users over the same experimental run to mitigate any inter/intra-operator influences. All coauthors were organ culture perfusion system operators. In IOP maintenance challenge experiments, an equal number of RS and D samples were cultured at the same time to mitigate effects caused by experimental run. Based on our perfusion system baseline IOP data from earlier work, a minimum sample size of 7 is required to detect an 18% change in IOP with an alpha of 0.05 and a power of 0.80 [31]. As our anterior segment perfusion culture and IOP measurement system can run eight eyes in parallel, this number was chosen, as in prior studies [24, 31, 53–55], for sufficient redundancy if a sample needed to be removed due to contamination or hardware error. IOP recordings were sampled into 2-h blocks, and IOPs were compared between treatment groups at each experimental phase (baseline, reseed/sham, and challenge). Periods of 8 h after media refilling were excluded from the analysis to allow for IOP stabilization. Statistical comparisons were conducted with a Student’s *t*-test in Python 3.6. Data are expressed as mean ± SEM unless otherwise noted. *p* values < 0.05 were considered statistically significant.

## Results

### Scaffold production

**Fig. 1:**
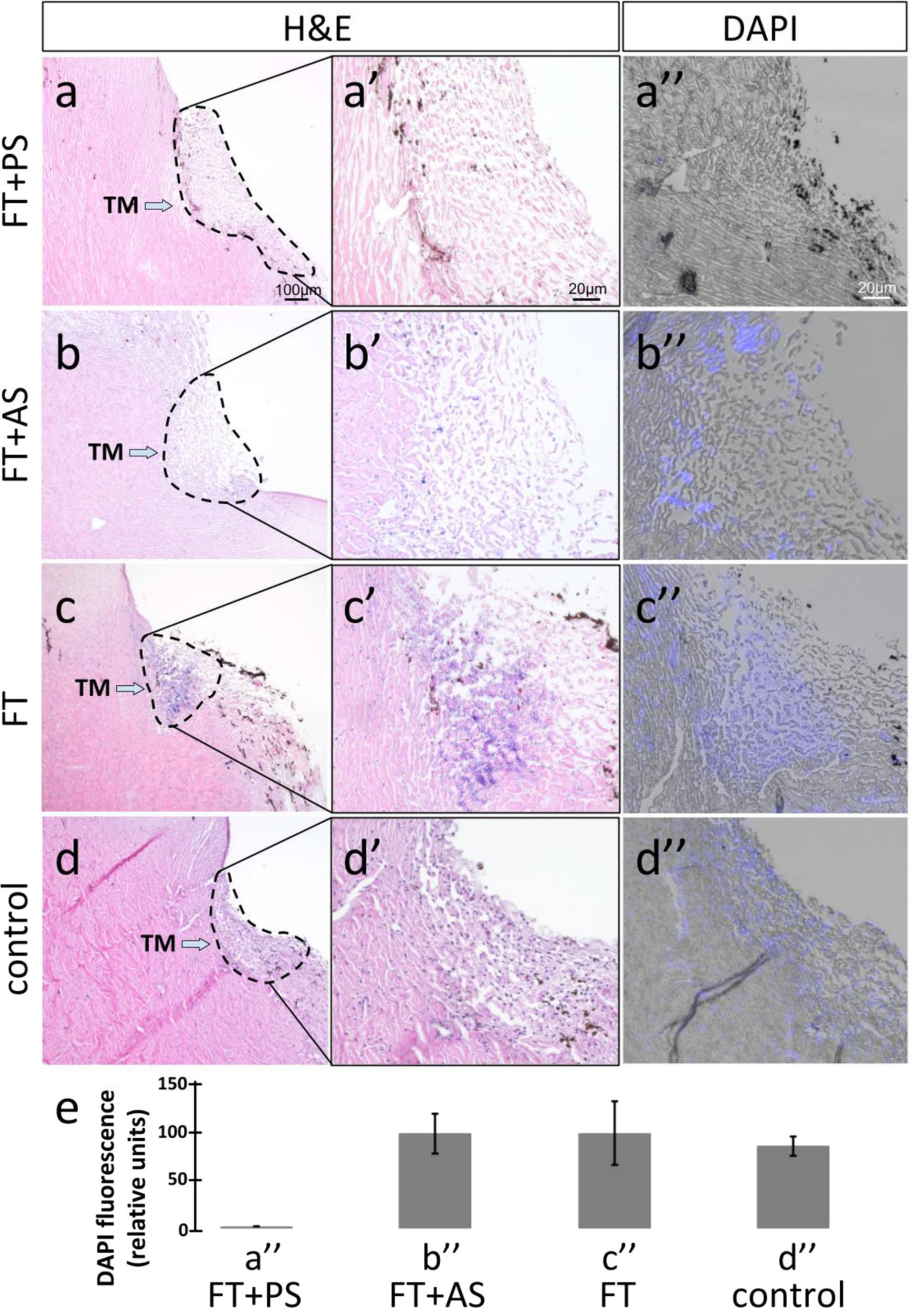
Left: H&E staining, middle: magnified view of TM. Right: DAPI + brightfield. (**a**) Freeze–thaw cycling and perfusion of surfactant (FT + PS) removed visible cells (a’) and nuclear debris (no blue DAPI staining (a’’)). (**b**) Cellularity was reduced after FT and agitation in surfactant (FT + AS (b’)), and nuclear debris was observed in distal TM (b’’). (**c**) FT alone destroyed cells, but nuclear material was present in mid and distal TM (c’ and c’’). (**d**) Untreated controls with normal TM cellularity (d’) and even, intranuclear DAPI staining pattern (d’’). (**e**) DAPI fluorescence was greatly reduced in FT + PS (a’’) but similar in groups FT + AS (b’’), FT (c’’), and controls (d’’)

After cycles of freeze–thaw and perfusion of surfactant (FT + PS, Fig. 1a, a’), no cells or nuclear debris could be detected by DAPI staining (Fig. 1a’’). The ECM structure was well-preserved. After FT and agitation in surfactant (FT + AS (Fig. 1b, b’)), some remnants of nuclear debris could be made out by DAPI staining in the TM most distal from the anterior chamber (Fig. 1b’’). FT + AS samples on days 1, 2, and 5 appeared similar. FT alone (Fig. 1c) destroyed cells but could show displaced nuclear material in the mid and distal TM (Fig. 1c’ and c’’). Untreated controls (Fig. 1d) had a normal TM cellularity (Fig. 1d’) and an even DAPI staining pattern. Here, DAPI was limited to nuclei within cells instead of the diffuse staining in FT + AS (b’’) and FT (c’’). Mean DAPI fluorescence in FT + PS was much lower than in controls (*p* = 0.010). Despite a different histological appearance as described, the mean DAPI fluorescence intensity was relatively similar in FT + AS (b’’), FT (c’’), and controls (d’’, FT + AS vs. controls: *p* = 0.575, FT vs. controls: *p* = 0.387). Because FT + PS had a well-preserved ECM but were without cells or nuclear debris, we used FT + PS scaffolds for all subsequent experiments.

IOP remained within a normal range (12.27 ± 0.04 mmHg, *n* = 15) throughout the perfusion-decellularization process without evidence of any abnormal physical stress on the ECM. After 24 h of washing, scaffold IOP remained stable (within a 0.6 mmHg range) through the remainder of the process. Washed scaffolds appeared grossly normal. Twenty-four scaffolds were produced. Eight IOP recordings were lost due to hardware errors. One eye was contaminated and not included in the IOP analysis.

### Scaffold repopulation and IOP

**Fig. 2:**
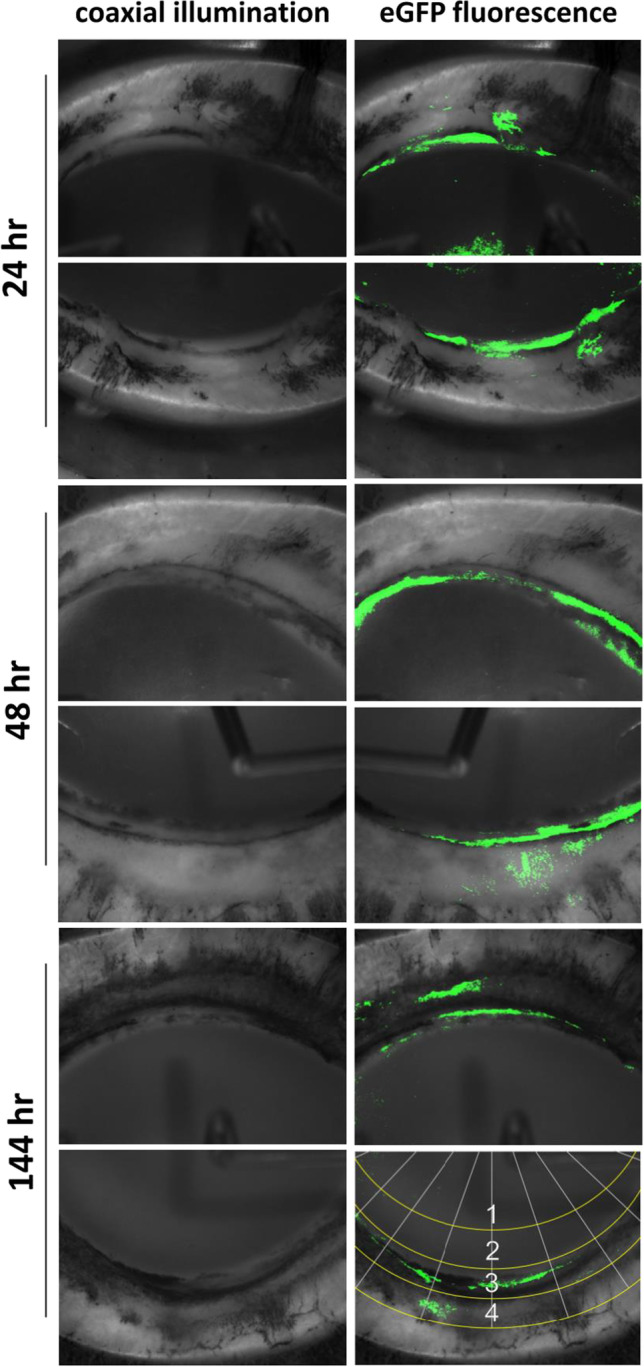
Anterior segment scaffolds seeded with 3 million eGFP CrFK cells were visualized directly through the underside of clear perfusion culture dishes. Seeded cells localized to the TM region. Left: brightfield view of TM angle, right: merged with eGFP fluorescence. Localization of intensity in fluorescence images was evaluated in the 4 concentric regions shown at the bottom right panel (central cornea (1), peripheral cornea (2), TM (3), and sclera/ciliary body remnant (4)). TM was brighter than all other regions (90.1 ± 7.5% brighter, *p* < 0.001)

We transduced robust CrFK cells with an eGFP-expressing lentiviral FIV vector, enriched them by fluorescence-activated cell sorting (FACS), and seeded them onto scaffolds to establish protocols for repopulation. The fluorescent signal of eGFP-expressing CrFK cells could be visualized at all time points (24, 48, and 144 h) and localized in the TM region. Occasional fluorescent cells could be seen on the corneal endothelium at 24 h but not at subsequent time points. Fluorescence was at times observed where the ciliary body had been attached. The TM was significantly brighter than other regions by an average of 90.1 ± 7.5% (*p* < 0.001, Fig. 2).

**Fig. 3:**
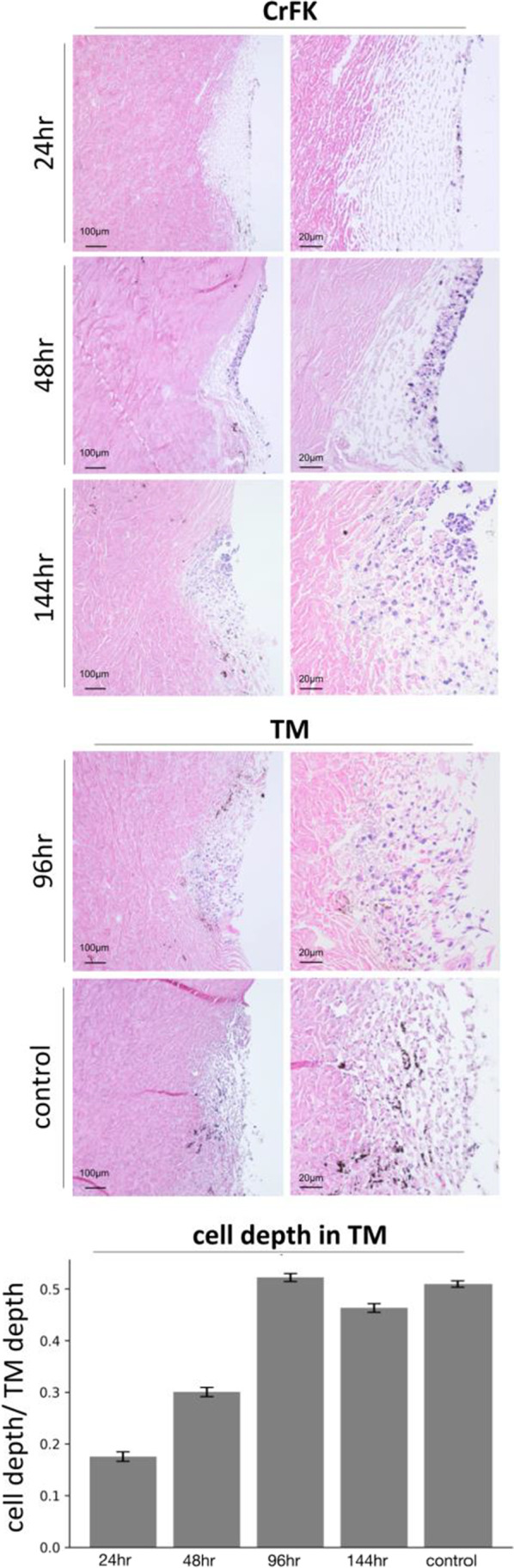
Cell infiltration of the TM at 24, 48, and 144 h for cultures reseeded with CrFK cells shown in Fig. 2. Cell infiltration of scaffolds reseeded with porcine TM cells is shown at 96 h. Cell depth at 24 and 48 h was significantly different from control (*p* < 0.001), while cell depth at 96 and 144 h and above was not significantly different (*p* = 0.19, 0.82). Error bars indicate SEM

The depth of cell infiltration into the TM was measured and compared to control at each time-point (supplemental material 1). The average depth of cells in the TM increased over time and peaked on day 4 (Fig. 3, Table 1). CrFK cell depth at 24 and 48 h was significantly lower than control (*p* < 0.05), while the depth of TM cells at 96 and CrFK cells at 144 h was not significantly different (*p* = 0.19, 0.06). At 24 h post-seeding, some cells could be identified histologically on the corneal endothelium, matching occasional cells fluorescing in Fig. 2. Cells migrated in the direction of outflow over time. Additionally, cellularity increased with time (Table 1, *n* = 3458 nuclei measured total). After 96 h in culture, many cells had large nuclei with euchromatin, indicating active transcription during infiltration. No cells were noticed in the region of the angular aqueous plexus, a region similar to Schlemm’s canal and proximal collector channels in primates [57].

**Table 1:** Cell infiltration of scaffolds

Time point	24 h	48 h	96 h	144 h	Control
Cell type	CrFK	CrFK	TM	CrFK	TM
Depth in meshwork (%)	17.5 ± 0.9*	30.0 ± 0.9*	52.2 ± 0.7	49.1 ± 0.7	50.9 ± 0.6
Cellularity (per section)	9.2 ± 1.6*	23.1 ± 4.4*	60.1 ± 6.4*	60.0 ± 13.0	100.0 ± 11.1

The depth of infiltrated cells is shown relative to the complete depth of the meshwork. Tissue-engineered TM cellularity values at each time point are normalized to control. Error indicates SEM. Asterisk indicates significant difference from respective control (*p* < 0.05)

**Fig. 4:**
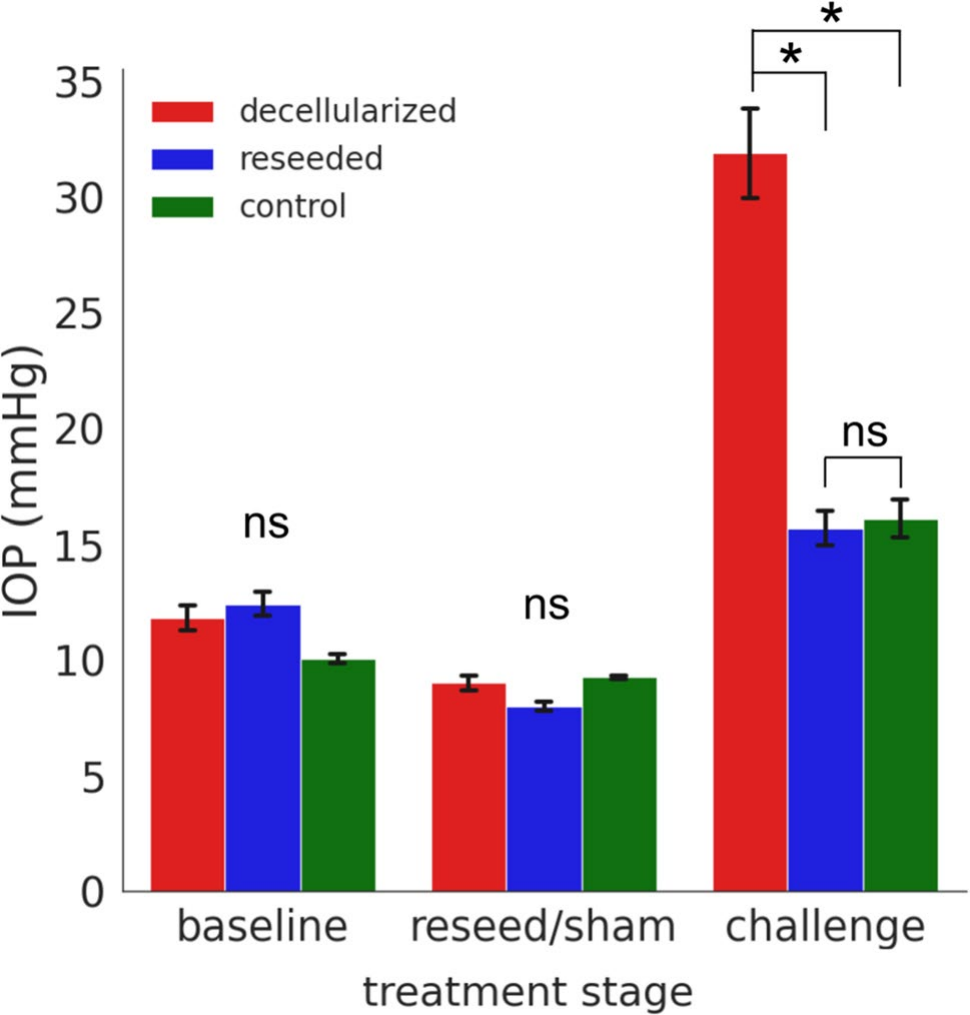
IOP of tissue-engineered ex vivo cultures. No significant difference was found between decellularized (red), reseeded (blue), and control (green) organ culture IOPs at the baseline or reseed/sham treatment stages. Reseeded scaffolds here received primary TM cells. For the IOP maintenance challenge, the infusion rate was doubled from 3 to 6 µL/min. Reseeded and control eyes maintained IOP within a normal range. IOP in decellularized scaffolds was significantly elevated in comparison to reseeded (*p* < 0.0001) and control (*p* = 0.009) organ cultures. *N* = 8 for decellularized and reseeded groups, *n* = 7 for control. Error bars indicate SEM, ns = not significant, *p < 0.01

Stable IOPs were achieved for each treatment group (decellularized (D), reseeded (RS), and control (C), *n* = 8) after 24 h in the ex vivo perfusion system. One eye in C developed a leak and had to be removed, reducing its count to *n* = 7. No significant differences were found between IOP of the D, RS, or C groups at baseline (D = 11.8 ± 0.5 mmHg, RS = 12.4 ± 0.5 mmHg, C = 10.1 ± 0.2 mmHg). There was no difference between IOPs after seeding group RS (D = 9.2 ± 0.4 mmHg, RS = 8.5 ± 0.2 mmHg, C = 9.2 ± 0.1). However, scaffolds seeded with CrFK cells had a higher IOP that was unstable, as indicated by a large standard deviation, a measure of the amount of variation (27.0 ± 17.3 mmHg (avg ± SD)) when compared to TM cells (8.5 ± 2.7 mmHg (avg ± SD)). At 48 h post TM reseeding in RS or sham procedure in D and C, the infusion rate was doubled from 3 to 6 µL/min to challenge the TM’s IOP maintenance response. RS maintained IOP within a normal range not different from eyes in C, while D became hypertensive (D = 35.2 ± 2.2 mmHg, RS = 16.5 ± 0.9 mmHg, C = 16.9 ± 0.9, Fig. 4). The *p* values for statistical comparisons of treatment groups at each experimental stage are included in Table 2.

**Table 2:** *p* values of statistical comparisons of IOP at each experimental time-point

Comparison	Baseline *p* value	Reseed/sham *p* value	Challenge *p* value
Control vs. decellularized	0.205	0.938	0.009*
Control vs. reseeded	0.0666	0.0961	0.907
Reseeded vs. decellularized	0.395	0.0731	7.5 × 10^−14^*

*p* values of less than 0.05 were considered statistically significant (*)

## Discussion

TM cells have been transplanted into living trabecular meshwork both ex vivo [58] and in vivo in the mouse [59, 60], but a downside of this approach is that the effect and behavior of transplanted cells cannot be distinguished from the existing ones. The effect overlap is especially problematic if one wants to generate, for instance, an ex vivo model of pseudoexfoliation or a specific glaucoma mutation [61]. For this reason, we developed a tissue-engineered anterior segment organ culture model of TM transplantation that demonstrates key physiological similarities with standard ex vivo and in vivo TM models but lacks any recipient cells. We also wanted to have cultures that are easy to generate and scale. Current in vitro and 2D perfusion cultures are harder to produce [19] and lack biofidelic cues from a physiological, multilayered, 3D environment, a hallmark of this tissue. We found that not only is generating the scaffolds straightforward, but the ability to store them at − 80 °C and install them rapidly adds flexibility to experimental design, and the ability to scale up that cannot be matched by human donor eyes.

TM cellularity, distribution, and density were similar to control organ cultures. After an infusion rate challenge, control anterior segments and those with repopulated TM maintained a physiological IOP. These results were like those observed in standard human anterior segment perfusion models with infusion rate doubling [11] and after IOP increase [62]. IOP in anterior segments with decellularized TM nearly tripled, resulting in pathologically high pressure. TM cell numbers normally decrease with age, especially in glaucoma [63, 64]. Although partially removing TM cells can reduce IOP temporarily [51], denuded beams can also fuse, which is associated with TM collapse, reduction of intertrabecular spaces for outflow, and pathologically elevated IOP [65–70]. The increased IOP after the perfusion challenge in decellularized scaffolds is likely caused by collapsing intertrabecular spaces. TM compression because of increased IOP can lead to narrowing and rarefaction of outflow pathways with a declining facility as established in bovine [71] and human eyes [72]. In contrast, when porcine [73–75] or human eyes [76] are subjected to ab interno trabeculectomy, which removes both TM cells and ECM beams, outflow reliably increases.

Abu-Hassan et al. [27] recently showed that the transplantation of human TM cells or TM-like iPSCs into the anterior segment perfusion model can restore outflow after killing about 1/3 of TM cells with saponin. In our study, all resident cells were removed to guarantee that any observed effect could only be caused by the transplanted cells but not by residual native ones. We washed cell debris out of scaffolds and avoided the use of saponin to allow for a healthy TM cell function. This model allowed us to isolate the effects of TM cells on outflow physiology in the TM region.

The TM has many mechanisms at its disposal to adjust outflow, including cytoskeletal and extracellular matrix changes. Important mediators include PGF2α, TGFβ, IL-1α/β, TNFα, nitric oxide, adenosine, and Rho kinase [9–13, 48], among others. As expected, in contrast to TM cells, CrFK cells (a feline-derived kidney cell line) could not maintain IOP within a physiological range even at a constant infusion rate of 3 µL/min. Instead, IOP was high and quite unstable, as evidenced large standard deviation, a measure of the amount of variation. We focused on establishing TM ablation and repopulation and did not investigate the mechanisms of IOP regulation in this model.

We found that cells infiltrated the TM to a depth comparable to resident cells of normal controls. In pilot studies not presented above, we seeded 1 million cells, a cell number closer to a young, healthy TM [64], but this resulted in a low tissue-engineered TM cellularity. This is consistent with cell loss percentages in other tissue-engineered organs [33, 77]. Seeded TM cells may require sufficient cell–cell contact early in the process of meshwork infiltration, as they cannot survive or proliferate otherwise.

Limitations of this study include a lack of confirmation of the predominant mechanism by which TM cells keep IOP within a normal range, which is beyond the scope of the current work. Additionally, cells of the TM are a heterogeneous population of uveal, corneoscleral, juxtacanalicular, and stem cells. For this reason, future characterization of TM cells in these scaffolds needs to include an analysis of gene expression and location to determine further similarities and differences between tissue-engineered cultures and normal anterior segments. We utilized porcine primary TM cells due to their availability and the established physiology they share with human TM [24, 78, 79]. Another limitation is the loss of seeded cells, a common problem in tissue-engineered organ cultures. Ott et al. reported that 46% of cells seeded into bioartificial heart culture were lost already within 20 m [33]. Improving cell retention, for example, through the use of polymer gels, could further enhance efficiency in our model [80]. In future studies, electron microscopy analysis of TM cells in scaffolds could provide important insights into the similarities and differences between the TM of tissue-engineered organ culture presented here and conventional organ culture.

In conclusion, we developed a fully decellularized porcine anterior segment scaffold and reseeded it with non-native meshwork cells. In these engineered ex vivo cultures, transplanted TM cells homed to the TM region, infiltrated the ECM, and maintained IOP during an infusion rate challenge, each a structure and function characteristic of this tissue.

These tissue-engineered ex vivo cultures demonstrated homing of transplanted cells to the TM region, infiltration of the ECM, and IOP maintenance ability after infusion rate challenge, all of which are hallmarks of ex vivo culture structure and function. The scaffolds may allow the testing of TM cell lines, iPSCs, or patient-derived TM cells without the need for human donor eyes.

## Supplementary Information

ESM 1 (DOCX 59.2 kb)

## Data Availability

The datasets generated and analyzed during the current study are available from the corresponding author upon reasonable request.
