# Supplementary figures and images for: Tissue-engineered anterior segment eye cultures demonstrate hallmarks of conventional organ culture
Source: Graefes Arch Clin Exp Ophthalmol. 2022 Dec 24;261(5):1359–68. doi: 10.1007/s00417-022-05915-z (PMC10148776; doi:10.1007/s00417-022-05915-z)

# **Supplemental material**

## ***Supplemental material 1: Map of materials and methods
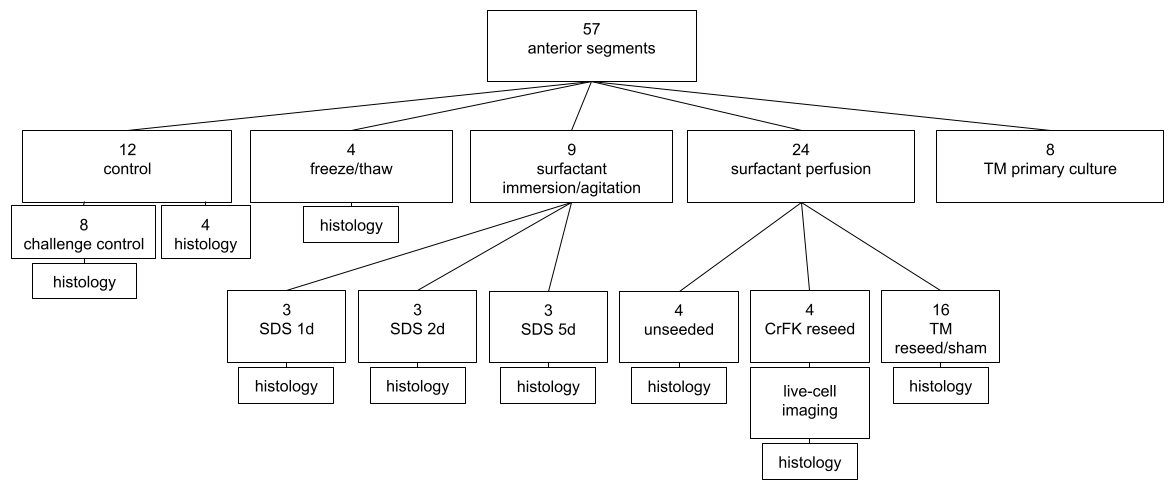
***

Supplement: Supplementary file 1 — (DOCX 59.2 kb) [file 417_2022_5915_MOESM1_ESM.docx]
